# Detecting Food Fraud in Extra Virgin Olive Oil Using a Prototype Portable Hyphenated Photonics Sensor

**DOI:** 10.1093/jaoacint/qsaa099

**Published:** 2020-07-22

**Authors:** Yannick Weesepoel, Martin Alewijn, Michiel Wijtten, Judith Müller-Maatsch

**Affiliations:** Wageningen Food Safety Research, P.O. Box 230, Wageningen, The Netherlands, 6700 AE

## Abstract

**Background:**

Current developments in portable photonic devices for fast authentication of extra virgin olive oil (EVOO) or EVOO with non-EVOO additions steer towards hyphenation of different optic technologies. The multiple spectra or so-called “fingerprints” of samples are then analyzed with multivariate statistics. For EVOO authentication, one-class classification (OCC) to identify “out-of-class” EVOO samples in combination with data-fusion is applicable.

**Objective:**

Prospecting the application of a prototype photonic device (“PhasmaFood”) which hyphenates visible, fluorescence, and near-infrared spectroscopy in combination with OCC modelling to classify EVOOs and discriminate them from other edible oils and adulterated EVOOs.

**Method:**

EVOOs were adulterated by mixing in 10–50% (*v/v*) of refined and virgin olive oils, olive-pomace olive oils, and other common edible oils. Samples were analyzed by the hyphenated sensor. OCC, data-fusion, and decision thresholds were applied and optimized for two different scenarios.

**Results:**

By high-level data-fusion of the classification results from the three spectral databases and several multivariate model vectors, a 100% correct classification of all pure edible oils using OCC in the first scenario was found. Reducing samples being falsely classified as EVOOs in a second scenario, 97% of EVOOs adulterated with non-EVOO olive oils were correctly identified and ones with other edible oils correctly classified at score of 91%.

**Conclusions:**

Photonic sensor hyphenation in combination with high-level data fusion, OCC, and tuned decision thresholds delivers significantly better screening results for EVOO compared to individual sensor results.

**Highlights:**

Hyphenated photonics and its data handling solutions applied to extra virgin olive oil authenticity testing was found to be promising.

Oil from the olive fruit (*Olea europaea sativa* L.) is mainly produced and consumed in the European Union. Most farmers, producers, and exporters of olive oil are located in Greece, Italy, and Spain, respectively. Olive oil, as part of the Mediterranean cuisine, has gained worldwide popularity. This led to shortages in production and an increase in fraud cases due to several vulnerabilities in the supply chain (1). In a recent fraud vulnerability study amongst 28 businesses [business-to-business (B2B) and retailers] in the extra virgin olive oil (EVOO) chain, it was shown that the lack of technical control measures such as fraud monitoring, tracking and tracing systems, and contingency plans is predominant (2). The main authenticity issues with olive oil were reported to be adulteration of olive oil with other edible oils or olive oils of less quality, as well as mislabeling of different olive oil quality grades and protected designations of origin (PDOs) (1). According to the EU Regulation No. 29/2012 (3), the category EVOO is a “superior category of olive oil obtained directly from olives and solely by mechanical means.” In contrast, olive oils labelled as “olive oil composed of refined olive oils and virgin olive oils (RVOO)” are “oils comprising exclusively olive oils that have undergone refining and oils obtained directly from olives” and the ones labelled as olive-pomace oil (OPO) contain “exclusively oils obtained by treating the product obtained after the extraction of olive oil and oils obtained directly from olives” or “exclusively oils obtained by processing olive pomace and oils obtained directly from olives”.

The development of effective technical control measures for olive oil is therefore a daunting task due to the large variety in products in the olive oil commodity. To cope with the complexity, many very sensitive, targeted analyses as well as low-cost portable fingerprinting analyses have been developed and implemented by standardization development organizations [i.e., International Olive Council (IOC), AOAC, ISO, etc.] and published in scientific literature (4–6). The targeted methods mainly focus on classes of components which indicate the different processing grades of the olive oils, for example by assessing the presence of monochloropropanediol (MCPD) esters as demonstrated by Yan et al. (7). However, especially for B2B and retailers, fast, low-cost, and universally applicable non-targeted screening methods are needed, such as approaches using miniaturized vibrational spectroscopy (8) or other innovative fast methods like the usage of pulsed ultrasound (9). Data generated from these fingerprinting methods is processed via multivariate statistics to discriminate between classes of oils or concentrations or discriminate one product from all other products by one-class classification (OCC) modelling. For the latter, all samples where abnormal fingerprints are observed may be selected for further in-depth analyses (10, 11). Recently, “AOAC *Standard Method Performance Requirements* (SMPRs) for Non-Targeted Testing (NTT) of Ingredients for Food Authenticity/Fraud Evaluation of Extra Virgin Olive Oil” have been drafted, defining the upper boundary for fraudulent admixtures at 5% (*v/v*) (12).

In addition to the shift towards fast, low-cost, and universally applicable analysis methods in food fraud detection, measurements are required to be performed on site in a non-invasive manner. This focusses the development towards the application of portable devices that carry miniaturized optical spectrometers (13–16). Spectroscopic approaches such as fluorescence (FLUO), ultra-violet (UV), visible (VIS), near- and mid-infrared (NIR, MIR), Raman, and nuclear magnetic resonance spectroscopy have been used in the past to identify adulterated olive oil (17). Some examples of the many applications of single photonics sensors showing promising results on EVOO classification were reported by Durán Merás et al. (18) using a FLUO spectrophotometer and Vanstone et al. (19) and Yan et al. (8) using NIR spectrophotometers. Nevertheless, all individual photonic technologies have accidental incorrect classifications, so-called false positives and false negatives. By combining photonic approaches in EVOO authentication, classification rates may be improved and the LOD for fraudulent admixing can be lowered. However, studies on the application of such hyphenated devices seem to be lacking. Hyphenated devices may carry  multiple sensors and technologies like spectrometers covering multiple wavelengths or Raman lasers within one device (14). The combination of the data, i.e., fusion of spectra or statistical output, is then believed to give a more accurate classification of the sample (10).

This study presents for the first time the application of a hyphenated device,”PhasmaFood”, developed and built during the EU-H2020 project to classify EVOO, other grades of olive oils, edible oils, and fraudulent additions to EVOO in transflection mode. The “PhasmaFood” device combines three spectroscopic approaches and an RGB-camera, to detect transflectance spectra of NIR, VIS, and fluorescent (FLUO) radiation. The data from different sensors were derived from the same spot at the same time on the same liquid sample present in the cuvette. A suitable data fusion method is presented in detail, the accuracy of fraud detection tested on multiple adulterated EVOOs and the accuracy of authenticity detection by discriminating wrong labelled EVOOs from authentic ones using OCC.

## METHOD

### Materials

Chloroform [99.9% (*w/w*)], glacial acetic acid [100% (*w/w*)], potassium iodide (for analysis), sodium thiosulphate (0.1 mol/L), and starch solution (for analysis) were purchased at Merck KGaA (Darmstadt, Germany). Isooctane (PEC grade, >99.5%) was from Actu-All Chemicals (Oss, The Netherlands).

### Sample Collection and Preparation of Adulterated Samples

Oil samples were purchased at local supermarkets and retailers in The Netherlands. Sixteen EVOOs, 32 olive oils composed of RVOOs, and nine OPOs originating from Italy, Spain, Greece, and Turkey were purchased. Twelve other edible oils, four rapeseed oils, three sunflower oils, hazelnut, walnut, rice, soy, and peanut oil were purchased. EVOOs were adulterated by volume-to-volume mixtures of 10, 25, and 50% with RVOOs, OPOs, and other edible oils chosen randomly to cover a wide range of possible mixtures. All samples described above were included in the fabricated admixtures, resulting in 20 EVOO mixtures with RVOOs and OPOs and 40 EVOO mixtures with other edible oils. In total, 129 unique samples were included in this study: 16 EVOO, 32 RVOO, 9 OPO, 12 other edible oils, and 60 adulterated EVOOs.

### Reference Methods for Testing of EVOO

EVOOs were tested for their peroxide index, K_232_, K_268_, and Delta-K levels in accordance with Regulation (EU) No. 2568/91 to determine and record the proportion of oxidized constituents in an olive oil (20). The determination of the peroxide value was done in accordance with Annex III of this regulation. The K_232_, K_268_, and Delta-K levels were performed according to Annex IX of the same regulation, using 4 mL cuvettes and a standard issue spectrophotometer (UV-Vis spectrophotometer Cary 300, Agilent, Santa Clara, CA, USA). In addition, extraction and analyses of fatty acid esters of 2-chloropropane-1,3-diol (2-MCPD), 3-chloropropane-1,2-diol (3-MCPD), and glycidol esters (GEs) were conducted according to AOCS Cd 29a-13 (21) with slight modifications as in Yan et al. (2018) (22) using GC-tandem mass spectrometry (MS/MS).

### Hyphenated Photonics Measurements Using the “PhasmaFood” Sensor

**Figure 1.**
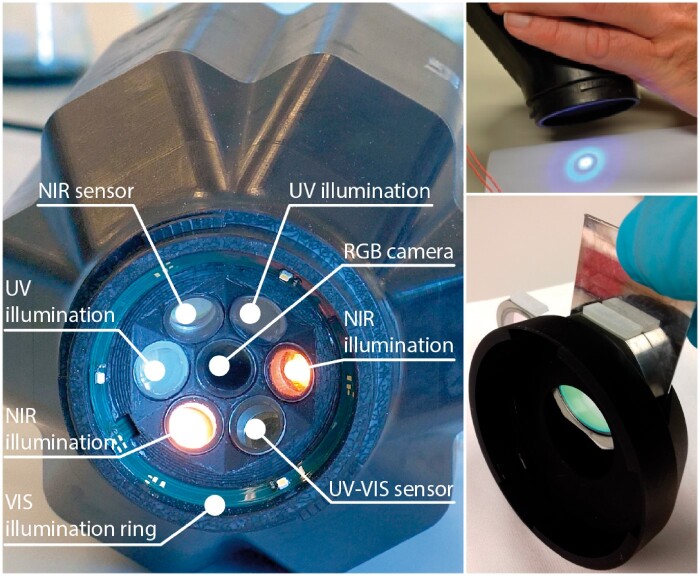
Pictures of the “PhasmaFood” device: (left) the sensing node, (top right) the handling of the prototype, (bottom right) the customized cuvette and cuvette holder for liquid samples.

Spectral data was acquired using a prototype portable, hyphenated, optical sensor, “PhasmaFood,” with serial number 001 fabricated by Fraunhofer IPMS (Dresden, Germany) and WINGS ICT solutions (Athens, Greece). The hyphenated sensor was equipped with a prototype miniaturized NIR sensor developed in-house [range 939–1833 nm, 895 individual wavelengths recorded, MEMS-type, Fraunhofer IPMS, patent no. WO 2003069289 A1, Pügner et al. (2016)], a miniaturized commercial UV-Vis spectrometer (range used 320–889 nm, 288 individual wavelengths recorded, C12880, Hamamatsu, Japan), and a miniaturized RGB-camera (MU9PC-MH, CMOS, Ximea, Münster, Germany). As depicted in Figure 1, the NIR and UV-Vis sensor front ends together with their respective light sources were positioned in a circular integrated setup containing a VIS light-emitting diode (LED) illumination ring, two 365 nm monochromatic lights for FLUO spectroscopy, and two NIR broad-spectrum lamps. The UV-Vis spectrometer was used for both the FLUO irradiance spectroscopy (365 nm irradiance) and the diffuse reflectance VIS spectroscopy. The RGB-sensor was positioned as the central sensor and was in this experiment setup solely used for measurement quality check purposes such as the detection of the presence of air bubble or other irregularities in the oil samples. All sensors were aligned to acquire spectral data from the same sample spot in an automated and sequential acquisition procedure. The “PhasmaFood” sensor was operated by a custom-build “PhasmaFood” Android application developed by VizLore Labs Foundation (Novi Sad, Serbia) communicating via a Bluetooth interface and spectral data was sent to an online cloud repository. The settings of the individual sensors were optimized for the oils considered in this work, i.e., no signal saturation of any sensor, acceptable signal-to-noise ratio. As this sensor is a prototype, individual details on settings cannot be given as they are not transferrable to other systems. Prior to sample measurement, a white reference calibration was conducted using a cuvette filled with ambient air positioned in the transflectance unit with the 99% diffuse reflectance standard (Figure 1). Prior to measurement of each sample an automatic dark reference was recorded. During one measurement run, 10 VIS, 10 FLUO, and 255 NIR spectra were acquired.

Samples were transferred to a custom build cuvette (5 mL) equipped with sapphire windows, with the front of the cuvette (i.e., directed towards the reflective surface of the sample holder) coated to prevent artefacts in NIR measurements (NIR II AR, Edmund Optics Ltd, York, UK). The “PhasmaFood” sensor node was then equipped with a customized cuvette holder with a 99% reflecting white inert material at the back end of the cuvette position to facilitate transflection (i.e., both reflection and transmission, Figure 1). Every sample was measured in triplicate on three different days over a period of 2 months. This way the “natural” degradation of EVOO, after being in contact with air (oxygen) and stored at room temperature over a normal household-usage period was included in the study. In total, nine measurements were obtained for each sample, leading to 1161 measurements (129 samples × 9) with 11 610 VIS, 11 610 FLU, and 296 055 NIR spectra. VIS and NIR spectral measurements were corrected for dark and 99% diffuse reflectance white standard spectral data, respectively. FLUO spectra were solely dark reference corrected.

### Multivariate Statistics

**Figure 2.**
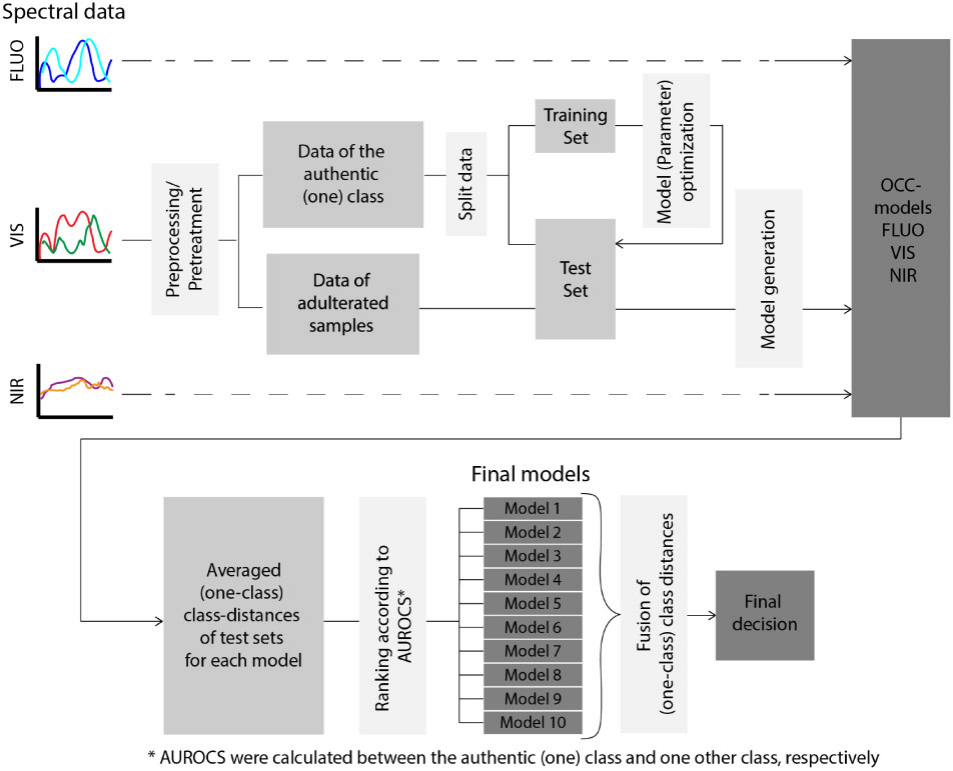
Spectral data processing, model generation, and optimization for one spectroscopic approach, evaluation of performance results, decision on final models, and data fusion.

All spectra acquired during one measurement run were preprocessed and averaged per measurement resulting in one sample spectrum per measurement run (1161 measurements in total). Data analyses were conducted using R version 3.6.1 (23). Areas of the spectrum containing irregular or noisy sensor responses were discarded leading to NIR spectra in the range of 1020–1833 nm (814 individual wavelengths), VIS spectra in the range of 400–740 nm (155 individual wavelengths), and FLUO spectra in the range of 340–780 nm (201 individual wavelengths). All FLUO and VIS spectra with saturated signals were detected visually as outliers and discarded (FLU 1161–122 = 1039 remaining spectra, VIS 1161–90 = 1071 remaining spectra). A total of 149 one-class models, using different data preprocessing steps and different chemometric algorithms (Table 1), were generated per sensor (spectral database) and optimized using internal cross-validation. Authentic class (EVOO) sample replicates were modelled using a data split of 0.8, where all (nine) scans of a sample were left out together (Figure 2A). All left-out EVOOs and all other scans were predicted and averaged, and their (one-class) class-distances were further evaluated. To select the one-class models for practical use, the strict one-class approach was abandoned, and the adulterated samples were included in the model selection. As depicted in Figure 2B, performance results from all models and sensors were ranked according to their area under the receiver operating characteristics (AUROC) values of the target (one-class) EVOO vs olive oils of lower quality, other edible oils, or adulterated EVOOs, respectively. A total of 10 models (six FLUO data models, two VIS data models, and two NIR data models) were selected manually from the available models based on these highest AUROCs. A high-level data-fusion approach was chosen, in which classification results from each individual sensor were combined. This high-level approach consisted of a decision tree methodology, i.e., if two or more out of 10 models classified a sample as “out-of-class” it was flagged as adulterated.

**Table 1.** Data preprocessing, spectral splitting, and chemometric algorithms applied for analysis of spectral data

Statistical approach	Name	Details	R package	References
Preprocessing	SNV	Standard normal variate	“prospectr”	(24)
	SNV detrend	SNV followed by baseline correction	“detrend”	(24)
	1st or 2nd derivative (Savitzky-Golay)	Derivative with 11-point filter length	“signal”	(25)
	Discrete wavelet transformation	Interpolation of the spectrum into 128 points; Application of discrete wavelet transformation, returning the 5th–7th level wavelet coefficients from a Daubechies with filter length 2 or the 3rd-5th level Least Asymmetric with filter length 8	“wavelets”	(26)
Spectral splitting		Spectrum split in 4 sections with equal lengths, each split being modelled separately		
Algorithms	SIMCA	Soft independent modelling of class analogies^a^	“mdatools”	(27)
	kNN	k-nearest neighbor^b^	“kknn”	(28)
	PCA residual	Principal components analysis residuals^c^		
	Mahalanobis distance	Calculated directly from the data using means and covariance of the training set		
	OCSVM radial kernel	One class support vector machine with radial basis kernel and automatic parameter estimation	“kernlab”	(29)

a Selecting the optimal number of components based on a five-fold (inner loop) cross validation.

b Selecting the optimal number of neighbors based on a five-fold (inner loop) cross validation.

c Calculating the sample residuals (Q residuals) using a selected number of PCs that were selected based on a five-fold (inner loop) cross validation.

## Results and Discussion

### Reference Values of EVOO Samples

EVOO samples were verified by peroxide index determination, K_232_, K_268_, Delta-K analysis using a benchtop spectrophotometer, and MCPD ester determination via GC-MS/MS (Table 2). Peroxide values are commonly used to rate the quality of olive oil. For the 16 EVOOs in this work, peroxide levels ranged between 12.25 and 55.48 mEq O_2_/kg (median 34.02 mEq O_2_/kg), where five EVOO samples were in accordance with the Regulation (EU) No. 2568/91  (20) with a peroxide index below or about 20 mEq O_2_/kg and 11 samples showing an indication of high peroxide value. It is believed that the high peroxide values resulted due to auto-oxidation of the olive oil, as the oils were transferred from the original bottles to glass bottles for storage before conducting the peroxide values. It is known that peroxide levels may increase rapidly after olive oil comes into contact with oxygen and that the maximum limit of 20 mEq O_2_/kg can be exceeded rapidly. Values then decline as peroxides are converted to secondary oxidation products. Therefore, this reference value by itself is no verification of the olive oils’ authenticity (30), and no samples were discarded based on these results. For further verification of the olive oils’ quality, spectroscopic investigations in UV (K_232_, K_268_, and Delta-K values, Table 2) were conducted. According to Matthäus (30), these reference measurements are of medium validity. The K_232_ results were within specifications for 14 samples, whilst for two samples the K_232_ values were above the set limit of 2.5 AU. As the K_268_ and the Delta-K of those samples were within specifications, we decided to retain the samples in the sample set. For the 3-MCPD, 2-MCPD, and GEs, results of all samples were in line with AOCS Cd 29a-13 (Table 2).

**Table 2.** Results of EVOO samples tested and their limits as described in Regulation (EU) No. 2568/91 and AOCS Cd 29a-13 for 3-MCPD, 2-MCPD, and GEs

Description of EVOO	Peroxide index, mEq O_2_/kg	K_232_, AU	K_268_, AU	Delta-K, -	3-MCPD, mg/kg	2-MCPD, mg/kg	GEs, mg/kg
Limit for EVOO	≤20.00	≤2.5	≤0.22	≤0.01	<0.10	<0.07	<0.07
Observed min	12.25	1.78	0.14	0.000	—^a^	—	—
Observed max	55.48	2.62	0.21	0.005	<0.10	<0.07	<0.07
Observed median	34.02	2.32	0.16	0.001	—	—	—

a — = Not available.

### Raw Spectral Data

**Figure 3.**
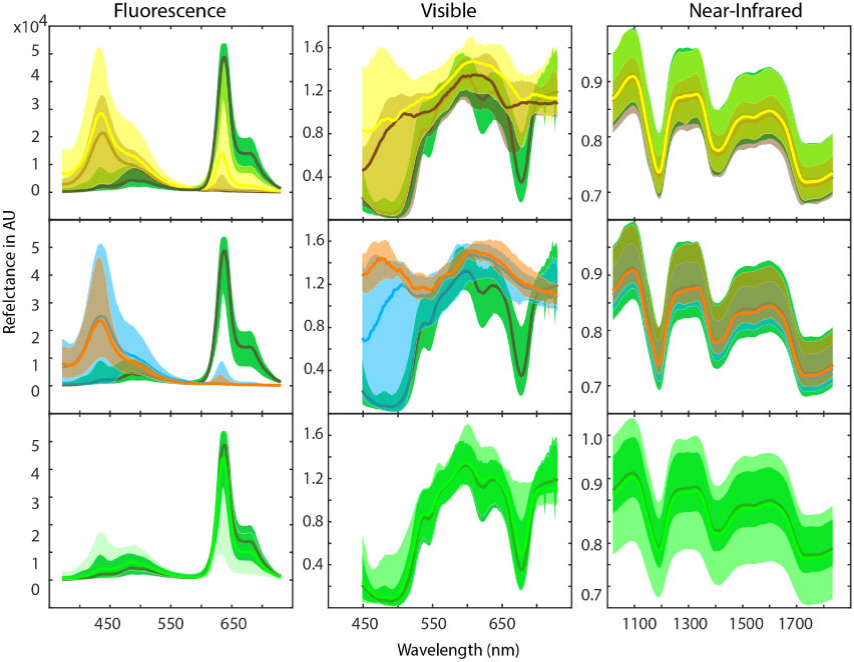
Reflectance spectra of FLUO, VIS, and NIR spectroscopy. Top row: EVOO (dark green) compared to refined olive oils (yellow) and olive-pomace oils (brown); Middle row: EVOO (dark green) compared to sunflower oil (orange) and other edible oils (blue); Bottom row: EVOO (dark green) compared to mixtures of EVOO with non-EVOO olive oils or other edible oils (lime). The bold spectral lines in the plots correspond to the respective median of all spectra in the respective area from minimum and maximum spectra.

The raw spectral data (dark- and, where applicable, white-corrected) for all three types of spectroscopy conducted are displayed in Figure 3. When comparing FLUO spectra of EVOO with RVOO and OPO, major differences in emittance concerning the spectral position and intensity of the Soret (wavelengths 400–500 nm) and the q-bands (600–700 nm) can be observed and have been extensively reported by Zandomeneghi et al. (31). The different quantities of porphyrin structures present in the oils, mainly being pheophytin *a* for olive oils, was the source of these observations (32). Depending on the country of origin, olive cultivar, and the EVOO production method, (traces of) chlorophylls *a* and *b*, pheophytin *a*, and pheophorbide *a* may be present. For the FLUO spectra this may be observed as shoulder peaks next to the main Soret band and q-band. For RVOO and OPO, losses and degradation of porphyrin structures may occur up to 80% of the amount present in EVOOs , resulting in a visually different FLUO spectrum. As this sample set contained EVOOs with a variety of geographic provenances, the number and quantity of porphyrin structures differed within the EVOO sample class (32). In the area of 350–400 nm, major differences can also be observed between EVOO, RVOO, and OPO, linked to the presence of carotenoid pigments, fluorescent phenols, and other miscellaneous compounds (31). Similar observations can be made for the sunflower and other edible oils, when comparing to EVOO in the wavelength areas of 400–500 and 600–700 nm. Differences between sunflower and other oils and EVOO are very pronounced around the q-band area. When mixing EVOO with the other oils up to 50% (*v/v*), the q-band remains the dominant fluorescence peak, followed by the Soret band and the band at 350–400 nm. Interestingly, the RVOO, OPO, and sunflower oil show high between-sample variance when comparing the minimum and maximum spectra, whilst the EVOO spectra seem to be relatively uniform. In conclusion, EVOO had a very distinct and high intensity FLUO spectrum which may be useful for classification of EVOO versus non-EVOO. However, when EVOO is mixed with other oils, the FLUO spectrum alone may not be suitable due to its high-intensity q-band.

Similar to the FLUO raw spectra, in the VIS spectra the pheophytin *a* pigment in the EVOOs was clearly observed in the Soret and q-band areas. OPO, RVOO, sunflower oil, and the other edible oils lacked this band. Furthermore, the intensely colored EVOO could, over the entire length of the VIS spectrum, be distinguished from the other edible oils. The latter commonly lack pigments due to their nature of refining by a clear spectral shape as was reported in literature (33). Upon mixing EVOOs, the VIS spectrum alone may as the FLUO one alone not be suitable for food fraud detection purposes, as mixing translucent oils cannot be detected.

The NIR spectra of the oils contained chemical information on the macro- and micro-compositions of the oils. As the oils have relatively similar macro-compositions, NIR spectra do not visually seem to contain information for distinguishing between different classes of olive oils or other oils. Multivariate statistics are required for conversion of NIR data to useful classification models to distinguish between EVOO, OPO, and RVOO. An extensive description of the chemical information confined in NIR spectra and OCC classification of EVOO, RVOO, and OPO can be found in the work of Yan et al. (2019) (34).

### Application of OCC to EVOO Spectral Data

The selected 10 OCC models yielded class distances for each spectrum predicted (Figure 2). For each sample, the triplicates (three different days over a period of 2 months) were combined and a decision threshold (decision tree) was applied to generate a fused OCC model delivering a final sample classification. In this work we considered two scenarios for the choice of this threshold: Scenario 1, where all EVOOs were classified correctly (100%, Table 3) and a more real-world practical scenario 2 where most admixtures of EVOOs were predicted “out-of-class” (97% and 91%, Table 4) at the expense of classifying some authentic EVOOs as “out-of-class” as well. In both scenarios, the RVOOs, OPOs, and other edible oils were classified correctly in 100% of the cases.

**Table 3.** Correct classification rates when applying threshold settings to avoid falsely identified EVOOs for the combination results obtained from the decision tree (scenario 1)

Sample	Combination (decision tree), %	Only FLUO,%	Only NIR, %	Only VIS, %
EVOO	100	86	100	80
RVOO	100	100	7	88
OPO	100	100	21	96
Other edible oils	100	100	39	94
Adulterated EVOOs with non-EVOO olive oils [10, 25, 50 % (*v/v*)]	76	81	8	83
Adulterated EVOOs with other edible oils [10, 25,50 % (*v/v*)]	65	66	18	54

**Table 4.** Correct classification rates when applying threshold settings to achieve an optimized identification rate for adulterated EVOOs for the combination results obtained from the decision tree (scenario 2)

Sample	Combination, decision tree, %	Only FLUO, %	Only NIR, %	Only VIS, %
EVOO	75	70	89	75
RVOO	100	100	37	99
OPO	100	100	50	100
Other edible oils	100	100	67	100
Adulterated EVOOs with non-EVOO olive oils [10, 25, 50% (*v/v*)]	97	97	31	83
Adulterated EVOOs with other edible oils [10, 25, 50% (*v/v*)]	91	89	52	56

In the first scenario, class decision scenarios were set such that no false negative classifications occurred, meaning that all EVOOs were correctly identified, as well as all pure RVOOs, OPOs, and other edible oils. However, scenario 1 has the tendency to yield false positives, classifying admixtures of EVOOs as authentic EVOOs (Table 3). Looking at the adulterated EVOOs (admixtures), classification errors significantly increased to unacceptable false positive levels of 24% (100–76%) and 35% (100–65%) for adulteration with non-EVOO olive oils and other oils, respectively. Therefore, scenario 1 is best applied for identification of raw materials and not as a screening method for routine applications when fraudulent additions might be encountered. Still, scenario 1 was very effective for correct classification (100%) of pure oils, as all were designated correctly as EVOO or non-EVOO. Considering the advantages of the hyphenated sensor approach, the OCC classification benefitted from the three-sensor approach, leading to an improved combined classification score (Table 3). Clearly, the information of the distinct FLUO spectra played an important role here followed by the VIS spectra (see also the section *Raw Spectral Data*). As the NIR spectra mainly concern macro-composition, it possibly played a negligible role in the combined classifications, as was reported before (8, 19).

**Figure 4.**
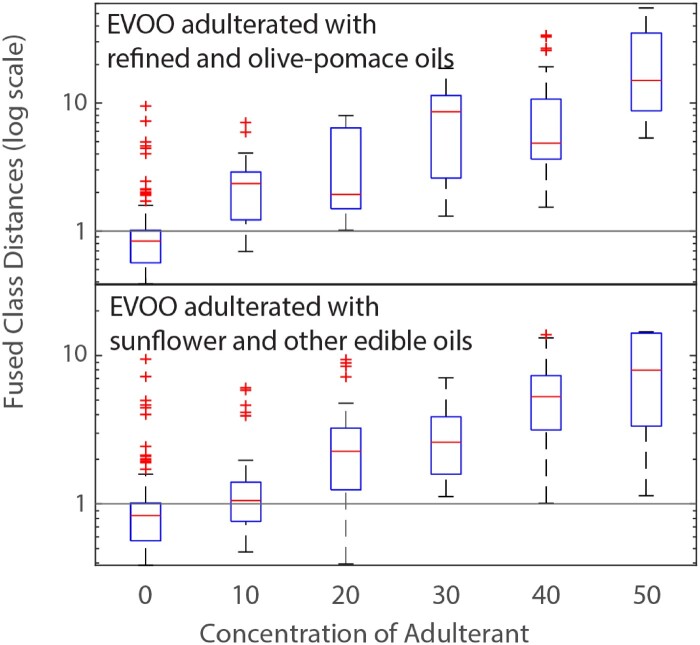
Fused class distances for EVOO adulterated with (top) RVOO and OPO and (bottom) sunflower and other edible oils.

From a practical fraud screening point of view, scenario 2 (Table 4) reduces the number of false positive classifications and is therefore more suitable for detection of fraudulent additions to EVOO. In this scenario, fraudulent admixing of EVOOs with non-EVOO olive oils or other oils was found correctly in 97 and 91%, respectively, for concentrations ranging between 10 and 50% (*v/v*). As a drawback, the correct classification of EVOO is reduced in scenario 2 to 75%. In Figure 4, the fraudulent additions to EVOO are displayed in more detail in increments of 10% (*v/v*) for scenario 2. Clearly, the success rate of fraud detection increased with increasing adulterant concentration. For EVOO adulterated with OPO and RVOO, all samples above 30% (*v/v*) adulteration were classified correctly (100%). Adulterations of 10 and 20% were more challenging, but the majority of adulterated samples were detected correctly. Classification of the EVOOs was generally successful, but big differences between EVOOs lead to a correct classification rate of 75%. For the adulteration with sunflower and other oils, a similar result was obtained, however the LOD is higher. Above 20% (*v/v*) adulteration, few adulterated samples appeared to score within the EVOO-one-class. At adulteration of 10% (*v/v*) the applied OCC method was not effective anymore. The collected data of the three sensors clearly did not contain enough information to distinguish a 10% (*v/v*) addition of refined plant oils from pure EVOOs.

By proposing two non-targeted OCC scenarios for the data from the hyphenated sensor, the “AOAC SMPRs for Non-Targeted Testing (NTT) of Ingredients for Food Authenticity/Fraud Evaluation of Extra Virgin Olive Oil” (12) can be approached. Though the draft SMPRs request more stringent demands on validation sample set size (270 samples over nine sample classes) and a minimum admixture percentage [5% (*v/v*) adulterant, correctly identified in 100% of the cases], the hyphenated sensor approach seems a viable and low-cost option to explore further. We do foresee that geographical provenancing and admixtures of falsely provenanced EVOOs, as stated by AOAC, will remain a challenge for both photonic as well as high-end analytics. The prototype 001 “PhasmaFood” was the first built sensor of its kind and many options for hardware optimization are still in signal-to-noise improvement. Also, by installing multiple excitation light sources for performing fluorescence spectroscopy, the amount of spectral data sets acquired by the UV-Vis sensor can be expanded easily. Finally, with the fast development in miniaturized photonics and the expansion of effective wavelength range, hyphenated photonic sensors may be able to meet future demands on EVOO authentication.

## Conclusions

For the first time a hyphenated photonic sensor, containing FLUO, VIS, and NIR, was used for the authentication of EVOOs by means of OCC modelling. It was shown that the combination of these three sensing solutions resulted in a benefit for classification of EVOOs and other oils and that it resulted in better detection of fraudulent additions to EVOOs. Of paramount importance, as demonstrated, is the application of data-fusion and OCC decision making. Depending on the specific situation where OCC is deployed in combination with hyphenated optics, a choice is necessary over the suitable decision threshold scenario. In this work we demonstrated two scenarios on classification of oils and identification of fraud with admixing of lower-cost oils to EVOO. Of course, many more scenarios can be thought of, or deployed simultaneously upon encountering an EVOO sample which is in need of screening.
